# A Rare Case of Low-Grade Bilateral Proximal Humerus Chondrosarcomas Managed With Staged Curettage and Cementation

**DOI:** 10.7759/cureus.80854

**Published:** 2025-03-19

**Authors:** Arın Celayir, Cansu Elibollar, Ahmet Burak Demirdas, Erdem Sahin, Ayse Mine Onenerk, Huseyin Botanlioglu

**Affiliations:** 1 Department of Orthopaedics and Traumatology, Istanbul University-Cerrahpasa, Cerrahpasa Faculty of Medicine, Istanbul, TUR; 2 Department of Pathology, Istanbul University-Cerrahpasa, Cerrahpasa Faculty of Medicine, Istanbul, TUR

**Keywords:** bilateral humerus tumors, curettage and cementation, enchondroma differential diagnosis, low-grade chondrosarcoma, proximal humerus cartilage tumors

## Abstract

Low-grade chondrosarcomas are rare malignant cartilaginous tumors with slow growth and low metastatic potential. Bilateral involvement, especially in the proximal humerus, is an exceedingly rare presentation. This report discusses a 45-year-old female patient who presented with intermittent left shoulder pain and was subsequently found to have bilateral proximal humeral masses on imaging. Bilateral contrast-enhanced MRI suggested a diagnosis of enchondroma or low-grade chondrosarcoma. The patient underwent curettage and cementation, first on the left side, followed by the right side four months later. Pathological analysis of the left lesion confirmed low-grade chondrosarcoma. During the second surgery, a frozen section revealed atypical chondromatous cells, with final pathology also supporting the diagnosis of low-grade chondrosarcoma. This case underscores the importance of careful diagnostic evaluation, including imaging and histopathological assessment, to differentiate low-grade chondrosarcoma from enchondroma. The multidisciplinary approach facilitated timely treatment, emphasizing the efficacy of curettage and cementation in managing these rare bilateral lesions while preserving function.

## Introduction

Low-grade chondrosarcoma is a rare, slow-growing malignant tumor originating from cartilaginous tissue. It represents a distinct category within the chondrosarcoma spectrum due to its relatively indolent behavior and low metastatic potential [1]. These tumors often pose diagnostic and therapeutic challenges, particularly when they present in atypical or bilateral locations. The proximal humerus, a common site for both benign and malignant bone tumors, is infrequently affected bilaterally by low-grade chondrosarcomas, making such cases noteworthy.

Clinically, low-grade chondrosarcomas often manifest with nonspecific symptoms, such as dull pain or discomfort, which may initially be misattributed to benign conditions [2]. Radiological imaging, particularly magnetic resonance imaging (MRI), plays a critical role in differentiating between benign lesions such as enchondromas and their malignant counterparts [3]. However, overlap in imaging characteristics often necessitates histopathological confirmation through biopsy or intraoperative tissue sampling, including frozen section analysis.

The differential diagnosis of low-grade chondrosarcomas includes enchondroma, a benign cartilaginous tumor that shares many overlapping imaging and histological features. Enchondromas are often asymptomatic and detected incidentally but may cause pain or pathological fractures if they enlarge or undergo malignant transformation [4]. Additional considerations in the differential diagnosis include bone cysts, giant cell tumors, osteoblastoma, and other cartilaginous tumors such as periosteal chondroma. Radiologically, distinguishing features such as cortical thinning, endosteal scalloping, and soft tissue extension may suggest malignancy, but definitive diagnosis often relies on identifying atypical chondromatous cells through histopathological examination.

Treatment typically involves surgical intervention, with curettage and cementation being standard approaches for low-grade lesions confined to bone [5]. This approach aims to achieve local control while preserving joint function, particularly in anatomically critical regions like the proximal humerus. Bilateral involvement adds a layer of complexity, requiring meticulous planning to minimize morbidity and optimize patient outcomes.

This report discusses the presentation, differential diagnosis, diagnostic process, and surgical management of a rare case of bilateral proximal humerus low-grade chondrosarcomas, highlighting the role of multidisciplinary tumor boards, the importance of pathological assessment, and the challenges associated with bilateral disease.

## Case presentation

A 45-year-old female patient presented to our clinic with complaints of intermittent pain in her left shoulder, which had gradually worsened over several months. She described the pain as more pronounced at night, interfering with her sleep, and unresponsive to conservative measures, including over-the-counter analgesics and rest. The patient had no known comorbidities and was not on any regular medication. Informed consent was obtained prior to any procedures.

Physical examination revealed a full range of motion in both shoulder joints, with no significant pain upon palpation. The absence of tenderness or functional limitations on examination contrasted with the patient’s reported symptoms. However, imaging was pursued due to the persistence and nocturnal nature of her pain.

**Figure 1:**
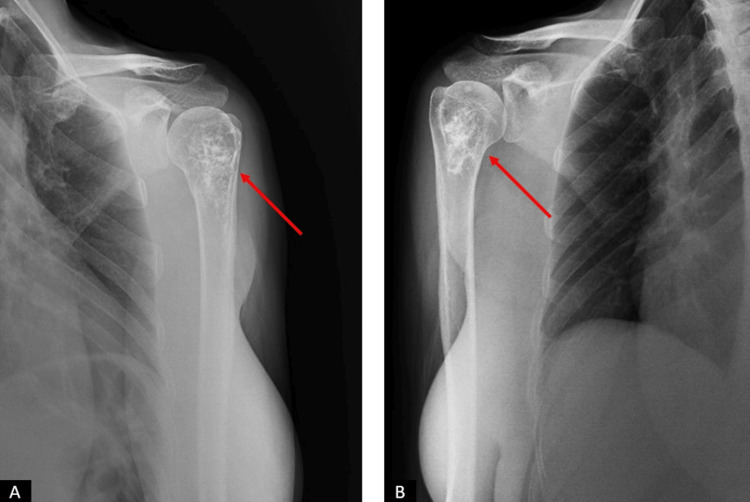
The patient's initial radiographs. (A) Left shoulder AP view. (B) Right shoulder AP view. The lesions in the humeral heads are indicated with red arrows.

**Figure 2:**
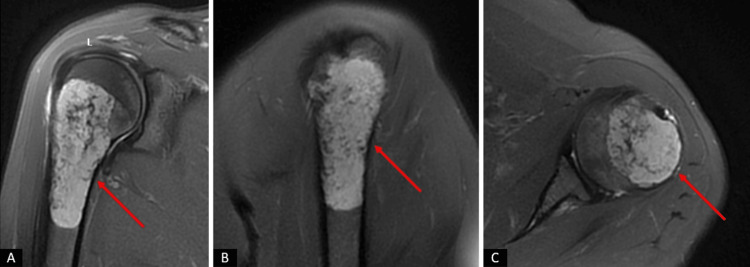
Preoperative magnetic resonance imaging (MRI) scans of the patient of left shoulder. (A) Coronal section MRI images of the patient. (B) Sagittal section MRI images of the patient. (C) Axial section MRI images of the patient. The regions in the proximal humerus with a preliminary diagnosis of enchondroma/low-grade chondrosarcoma are indicated with red arrows.

**Figure 3:**
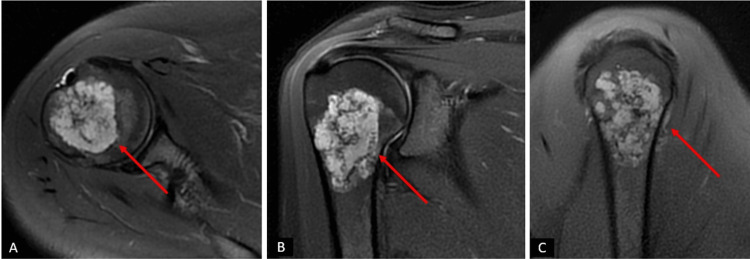
Preoperative magnetic resonance imaging (MRI) scans of the patient of right shoulder. (A) Axial section MRI images of the patient. (B) Sagittal section MRI images of the patient. (C) Coronal section MRI images of the patient. The regions in the proximal humerus with a preliminary diagnosis of enchondroma/low-grade chondrosarcoma are indicated with red arrows.

Radiological examination, including plain radiographs and bilateral contrast-enhanced shoulder MRI, revealed well-defined masses in the proximal humerus bilaterally (Figures 1-3).

The imaging characteristics suggested a preliminary differential diagnosis of enchondroma versus low-grade chondrosarcoma. Given the bilateral involvement and atypical presentation, the case was discussed in a multidisciplinary tumor board meeting, where curettage and cementation were deemed the most appropriate course of action.

**Figure 4:**
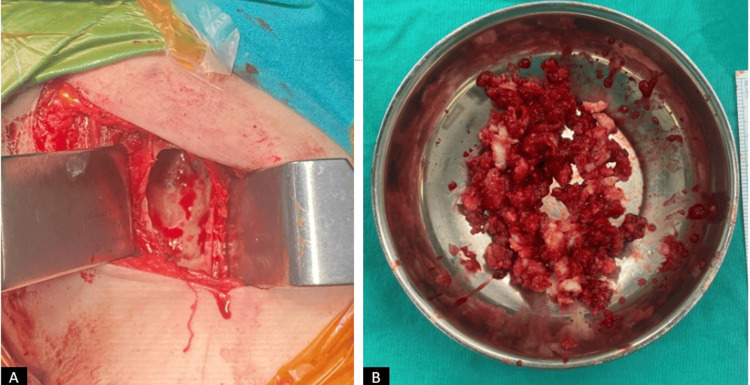
Intraoperative clinical images of the patient. (A) View of the proximal left humerus after creating a bone window. (B) Contents of the curetted material.

The patient first underwent curettage and cementation on the left side, which was the symptomatic side. During surgery, a bone window was created, and intramedullary tissues were curetted and sent for pathological examination (Figure 4).

The patient had an uneventful postoperative recovery, with no wound complications. Histopathological examination of the curettage specimen confirmed a low-grade chondrosarcoma.

**Figure 5:**
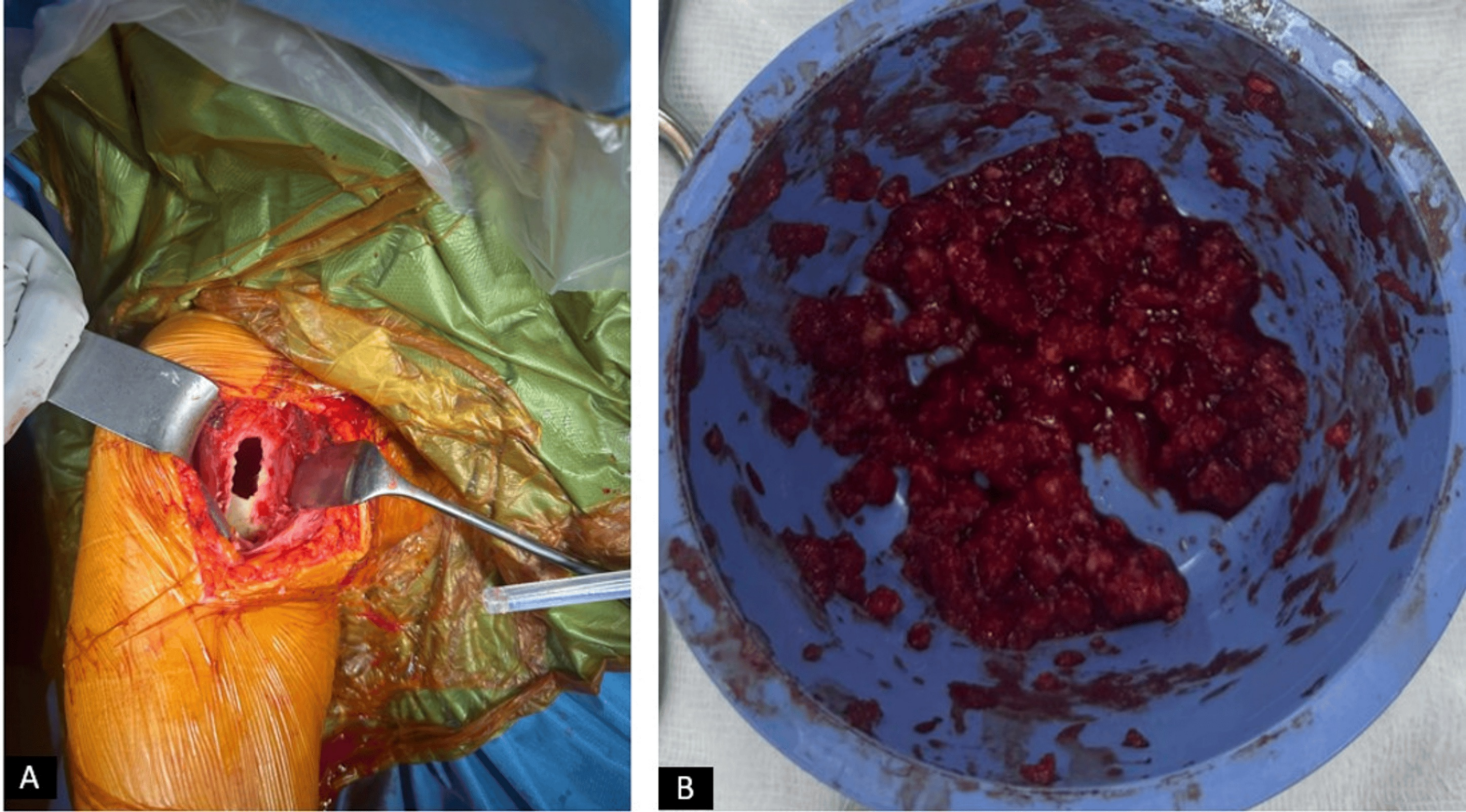
Intraoperative clinical images of the patient. (A) View of the proximal right humerus after creating a bone window. (B) Contents of the curetted material.

Four months later, due to persistent concerns regarding the lesion on the right side, the same procedure was planned for the contralateral humerus. During the second surgery, intraoperative frozen sections were sent for immediate evaluation (Figure 5).

**Figure 6:**
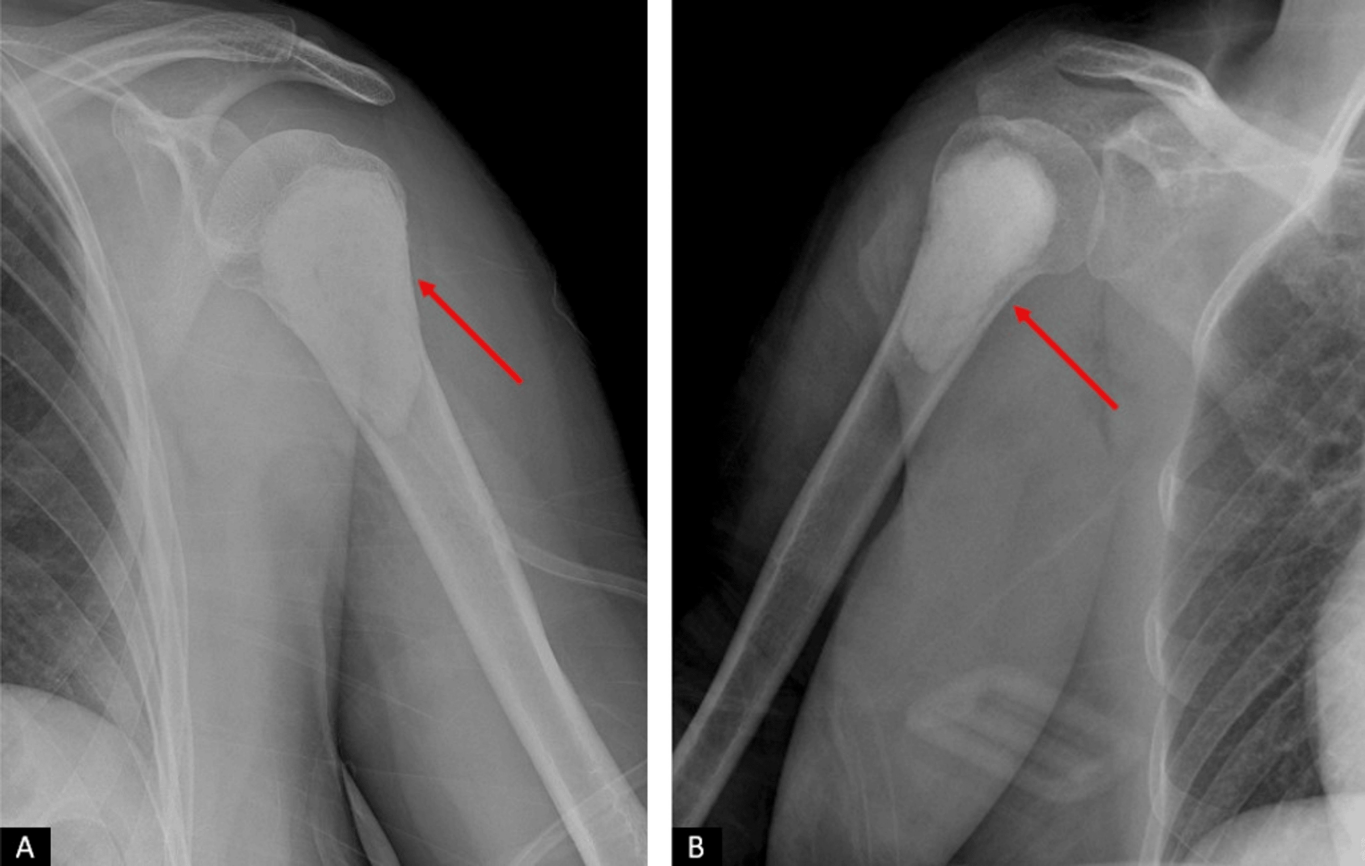
Postoperative radiographs of the patient. (A) Left shoulder AP view. (B) Right shoulder AP view. The bone regions where curettage and cementation were performed are indicated with red arrows.

The frozen section findings revealed atypical chondromatous cells, consistent with a low-grade chondrosarcoma. Curettage and cementation were successfully performed, and the patient tolerated the procedure well. The patient's postoperative X-rays were obtained, showing no pathological findings (Figure 6).

**Figure 7:**
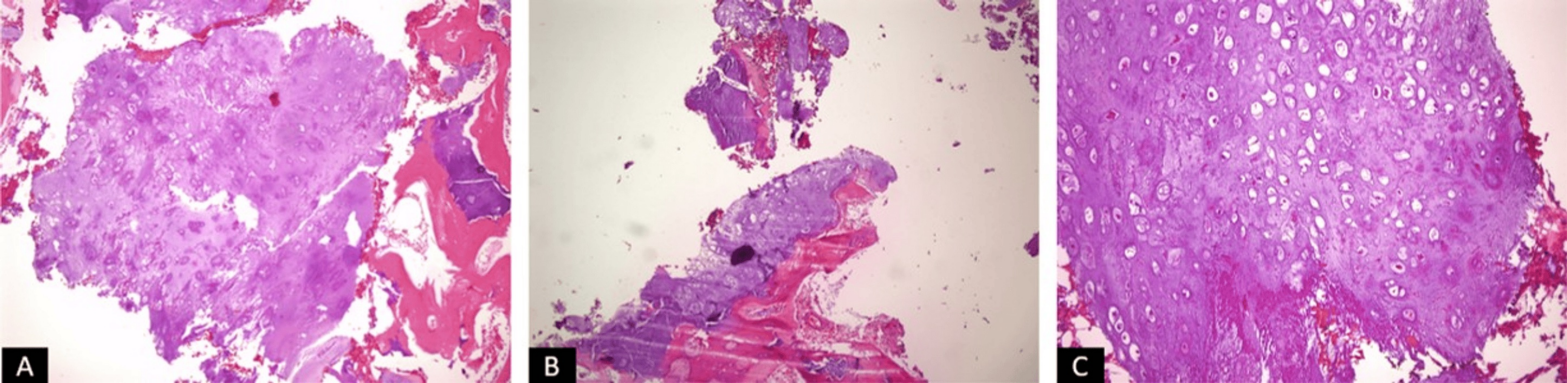
The specimen images from the microscopic examination of the pathological samples sent intraoperatively, stained with Hematoxylin and Eosin (H&E). (A-B) Images at x100 magnification. (C) Image at x200 magnification.

The patient experienced significant relief from pain postoperatively and was able to resume daily activities without limitations. The pathological examination of the surgical specimen was also consistent with low-grade chondrosarcoma (Figure 7).

Follow-up plans included regular clinical and radiological assessments to monitor for recurrence or progression. This case highlights the challenges of managing bilateral proximal humerus low-grade chondrosarcomas and emphasizes the importance of a multidisciplinary approach, particularly in cases with atypical presentations and refractory symptoms.

## Discussion

Low-grade chondrosarcomas are rare, slow-growing malignant tumors that arise from cartilaginous tissue and are most commonly found in long bones, including the proximal humerus [6]. Their indolent nature often leads to delayed diagnosis, as they may present with nonspecific symptoms such as intermittent pain or discomfort, as seen in our patient. Bilateral involvement of the proximal humerus is exceedingly rare and adds complexity to both diagnostic and therapeutic approaches.

One of the major challenges in managing low-grade chondrosarcoma lies in distinguishing it from benign cartilaginous lesions such as enchondromas. Both conditions can share overlapping clinical and radiological features, including cortical thinning, endosteal scalloping, and cartilage matrix mineralization [7]. However, key features such as persistent pain unrelated to activity and evidence of cortical disruption or soft tissue extension may raise suspicion of malignancy. Advanced imaging, particularly MRI with contrast, provides valuable insights, but the definitive diagnosis relies on histopathological examination. In this case, the diagnosis was confirmed through pathological evaluation of the excised tissues, which revealed atypical chondromatous cells consistent with low-grade chondrosarcoma.

The treatment approach for low-grade chondrosarcoma aims to achieve local control while preserving the structural integrity and function of the affected bone. Unlike high-grade chondrosarcomas, which require wide resection, low-grade tumors are often amenable to curettage and cementation [8-9]. This method allows for effective local control with minimal morbidity. In our case, the patient underwent staged bilateral procedures, first addressing the symptomatic left side and subsequently the right side. The use of intraoperative frozen sections during the second surgery enabled real-time pathological assessment, confirming the presence of atypical chondromatous cells and guiding appropriate management.

Ollier disease and Maffucci syndrome are rare disorders characterized by multiple enchondromas, with Maffucci syndrome also presenting with soft tissue hemangiomas. These conditions have a higher risk of malignant transformation into chondrosarcoma, often involving multiple skeletal sites. Unlike our case, which presented with isolated bilateral low-grade chondrosarcoma of the proximal humerus without any syndromic association, patients with Ollier disease or Maffucci syndrome typically exhibit multifocal and asymmetric bone involvement from an early age. The absence of systemic findings or additional skeletal lesions in our patient suggests that this case represents a sporadic occurrence rather than a syndromic manifestation, making it an unusual presentation of bilateral low-grade chondrosarcoma [10].

The decision to treat the lesions sequentially, rather than simultaneously, reflects a strategic approach to minimize surgical morbidity and ensure optimal recovery. Postoperatively, the patient experienced no early complications and functional outcomes were preserved, highlighting the efficacy of this approach. The importance of a multidisciplinary tumor board cannot be overstated, as it facilitates comprehensive evaluation and personalized treatment planning.

This case also underscores the importance of long-term follow-up in patients with low-grade chondrosarcoma. While the risk of metastasis is low, recurrence rates can range from 10-20%, particularly in inadequately treated cases. Regular imaging and clinical evaluation are essential for detecting any signs of recurrence or disease progression. MRI remains the preferred modality for assessing marrow involvement and soft tissue extension due to its superior contrast resolution.

## Conclusions

In conclusion, this case highlights the diagnostic and therapeutic challenges of bilateral low-grade chondrosarcomas of the proximal humerus. A multidisciplinary approach, combining advanced imaging, histopathological assessment, and staged surgical intervention, is crucial for achieving favorable outcomes. The successful management of this patient underscores curettage and cementation as an effective treatment strategy, balancing functional preservation with oncological control.

## References

[1] Karpik M, Reszeć J (2018). Low grade chondrosarcoma - epidemiology, diagnosis, treatment. Ortop Traumatol Rehabil.

[2] Biondi NL, Tiwari V, Varacallo MA (2024). Enchondroma. StatPearls.

[3] Weinschenk RC, Wang WL, Lewis VO (2021). Chondrosarcoma. J Am Acad Orthop Surg.

[4] Omlor GW, Lohnherr V, Lange J (2019). Outcome of conservative and surgical treatment of enchondromas and atypical cartilaginous tumors of the long bones: retrospective analysis of 228 patients. BMC Musculoskelet Disord.

[5] Ferrer-Santacreu EM, Ortiz-Cruz EJ, Díaz-Almirón M, Pozo Kreilinger JJ (2016). Enchondroma versus chondrosarcoma in long bones of appendicular skeleton: clinical and radiological criteria-a follow-up. J Oncol.

[6] Shemesh SS, Pretell-Mazzini J, Quartin PA, Rutenberg TF, Conway SA (2019). Surgical treatment of low-grade chondrosarcoma involving the appendicular skeleton: long-term functional and oncological outcomes. Arch Orthop Trauma Surg.

[7] LaPrade CM, Andryk LM, Christensen JL (2023). Natural history of intraosseous low-grade chondroid lesions of the proximal humerus. Front Oncol.

[8] Andreou D, Ruppin S, Fehlberg S, Pink D, Werner M, Tunn PU (2011). Survival and prognostic factors in chondrosarcoma: results in 115 patients with long-term follow-up. Acta Orthop.

[9] Gazendam A, Popovic S, Parasu N, Ghert M (2023). Chondrosarcoma: a clinical review. J Clin Med.

[10] El Abiad JM, Robbins SM, Cohen B, Levin AS, Valle DL, Morris CD, de Macena Sobreira NL (2020). Natural history of Ollier disease and Maffucci syndrome: Patient survey and review of clinical literature. Am J Med Genet A.

